# Downregulation of S100A9 Reverses Cisplatin-Resistance and Inhibits Proliferation and Migration in Hypopharyngeal Carcinoma

**DOI:** 10.1155/2022/9341731

**Published:** 2022-08-29

**Authors:** Shiyu Zeng, Guolin Tan, Tiansheng Wang, Xianyao Wang, Zheng Zhou, Jian Xiao, Tieqi Li, Gehou Zhang, Wei Li

**Affiliations:** Department of Otolaryngology-Head Neck Surgery, The Third Xiangya Hospital of Central South University, Changsha, Hunan Province 410013, China

## Abstract

**Purpose:**

Patients with hypopharyngeal carcinoma (HPC) often progress to an advanced clinical stage at diagnosis. Cisplatin has been widely used in first-line chemotherapy for advanced HPC. However, acquired chemotherapeutic resistance leads to recurrence, metastasis, and a poor survival rate. Therefore, identifying new drug targets to improve treatment effects is still in need.

**Methods:**

To screen the differential expression genes (DEGs) and proteins (DEPs), we conducted transcriptomic and proteomic analysis on cisplatin-sensitive cell lines (FaDu) and cisplatin-resistant cell lines (FaDu/DDP) of hypopharyngeal carcinoma. DEGs and DEPs, possibly the most associated with cisplatin-resistance, were verified by real-time polymerase chain reaction (RT-PCR) and western blot (WB), respectively, and the biological function of the screened S100A9 was further tested by CCK8, wound healing, and transwell assays.

**Results:**

We identified S100A9 as a target for resensitizing the response to cisplatin in an acquired resistance model. S100A9 overexpression was significantly related to cisplatin resistance. Functional studies in vitro models demonstrated that downregulation of S100A9 overcame cisplatin-resistance and inhibited proliferation and migration. Later, we verified that downregulation of S100A9 suppressed the interleukin-6 (IL6) expression and epithelial-mesenchymal transition (EMT) pathway.

**Conclusion:**

In all, S100A9 plays a crucial role in cisplatin-resistance, proliferation, and migration of HPC. Targeting S100A9 may become a novel strategy for the treatment of HPC.

## 1. Introduction

HPC is relatively rare, representing approximately 3% of head and neck cancer [1, 2]. Of these malignancies, squamous cell carcinoma of the hypopharynx comprises greater than 95% of HPC [3]. However, hypopharyngeal squamous cell carcinoma (HPSCCa) is characterized as the worst prognosis of head and neck cancer since the anatomy of the hypopharynx is rich in lymphatics and allows insidious growth until the symptoms present from invasion and metastasis. Most HPC often presents at an advanced stage. Compared to patients with early-stage who receive surgery and/or radiotherapy as standard treatment, advanced-stage patients benefit from chemotherapy [4]. Just like most malignancies, HPSCCa is sensitive to chemotherapy at the beginning. Unfortunately, drug resistance invariably emerges in the end.

Cisplatin is one of the best and widely used first-line treatments for solid cancers. It is used as the mainstay of chemotherapy drugs in combination with other drugs, especially in advanced HPSCCa [5]. Studies have shown that cisplatin exerts its anticancer activity by binding to genomic DNA or mitochondrial DNA, resulting in damaging DNA, ceasing DNA, mRNA and protein production, interfering with DNA replication, and activating several signaling pathways, eventually causing necrosis or apoptosis [6–9]. But the generation of drug resistance limits its use as a long-term treatment. Multiple molecular mechanisms of cisplatin-resistance in cancer have been demonstrated in others' research, such as DNA damage repair, suppression of apoptosis, overexpression of ATP-binding cassette transporters, and epigenetic regulation by miRNAs [10–12]. However, studies have not yet pointed to a particular approach to overcome the problem of drug resistance, which remains a challenge for us to identify therapeutic targets to address drug resistance in HPC.

S100 protein is named for its solubility in saturated ammonium sulfate solution. S100 protein family is a group of low molecular weight proteins that consists of 25 closely related members [13], and it is the biggest subfamily of calcium-binding proteins with EF-hand [14]. S100A9 is one of the S100 protein family. According to previous studies, S100A9 was found to be upregulated in esophageal, gastric, colon, pancreatic, bladder, ovarian, breast, thyroid, and skin cancers [15]. It has been experimentally demonstrated that S100A9 plays a part in the occurrence of inflammation and stimulates the release of inflammatory factors [16]. Recent investigations have demonstrated that S100A9 is involved in the proliferation, migration, and metastasis of various cancers [17]. Therefore, we are specifically interested in the role that S100A9 plays in the biological properties and drug resistance of HPC.

In this study, we cultured an HPC cell line-FaDu and established a cisplatin-resistant cell line-FaDu/DDP. We analyzed the DEGs and DEPs between FaDu and FaDu/DDP cell lines through transcriptomic and proteomic analyses. The remarkable overexpression of the S100A9 gene in the FaDu/DDP cell line motivated us to test whether S100A9 is an essential factor in promoting cisplatin-resistance in HPC. Here, we reported that S100A9 could lead to cisplatin-resistance in HPC and enhance the abilities of proliferation and migration meanwhile.

## 2. Materials and Methods

### 2.1. Cell Cultures

FaDu cell line was obtained from BeNa Culture Collection (Suzhou, Jiangsu, China, #BNCC316798). The FaDu/DDP cell line was built by slowly increasing the concentration of cisplatin (Sigma-Aldrich, St. Louis, MO, USA) exposed to parental cells [18]. Both cell lines were cultured by minimum essential medium (KeyGEN BioTECH, Jiangsu, Suzhou, China) containing antibiotics (80 U/ml penicillin G and 0.08 mg/ml streptomycin) and 10% fetal bovine solution (Hyclone, Logan, UT, USA) in a humidified atmosphere at 37°C with 5% CO_2_.

### 2.2. RNA Sequencing (RNA Seq) and Analysis

FaDu and FaDu/DDP cells were sent for RNA Seq, each with three replicates. Total RNA was extracted by TRIzol reagent (Thermo Fisher Scientific, Waltham, MA, USA). Concentration and qualification of isolated RNA tested by NanoDrop 2000 (Thermo Fisher Scientific). Sequencing libraries were prepared by KAPA Stranded RNA Seq Library Prep Kit (Illumina, San Diego, CA, USA) according to the suggested protocol of Illumina. Samples were sequenced by Illumina HiSeq4000 using the manufacturer's instructions. For data analysis, Solexa Pipeline (version 1.8) was used for image processing and base identification. Discard low-quality reads based on their quality checked by Fastp (version 0.12.5) [19]. All clean reads were aligned to reference human genome (GRCh37, hg19). The number of reads mapped to each gene was counted by HISAT2 (version 2.2.1) [20].

### 2.3. Tandem Mass Tag (TMT) Quantitative Proteomic Analysis

Proteins were extracted from FaDu and FaDu/DDP cells to obtain a peptide solution by filter-aided sample preparation. The pooled peptides were fractioned by reversed-phase chromatography. The peptide mixture was diluted with buffer A (0.1% formic acid) and loaded onto an XBridge Peptide BEH C18 Column (Thermo Fisher Scientific). The peptides were eluted with a linear gradient of buffer B (80% acetonitrile and 0.1% formic acid) at a flow rate of 1 ml/min. LC-MS/MS analysis was performed on Q Exactive HF-X mass spectrometer (Thermo Fisher Scientific) that was coupled to Easy nLC (Thermo Fisher Scientific) for 90 min.

### 2.4. GO and KEGG Analysis and Construction of PPI Networks

GO (Gene Ontology) and KEGG (Kyoto Encyclopedia of Genes and Genomes) pathways were analyzed by clusterProfiler R package [21–23]. GO classified DEGs and DEPs into three domains. GO terms with corrected *p* value less than 0.05 were considered significantly enriched by DEGs and DEPs. Pathways were arranged in the order of enrichment factor.

The STRING (https://string-db.org/) online database is used to explore internal interactions between DEGs and DEPs [24]. Cytoscape software is used to generate the protein-protein interaction (PPI) networks [25]. CytoHubba plugin is applied to get topological parameter value [26].

### 2.5. Real-Time Polymerase Chain Reaction (RT-PCR)

RNA was extracted by E.Z.N.A. Total RNA Kit (Omega Bio-Tek, Shenzhen, Guangzhou, China). The concentration of isolated RNA was tested by NanoDrop 2000. Reverse transcription was conducted by PrimeScript RT Master Mix (TaKaRa, Tokyo, Japan). RT-PCR was performed by SYBR Green Realtime PCR Master Mix (TaKaRa). The reaction conditions referred to the manufacturer's instructions. The expression of mRNA was normalized by GAPDH. The sequence of primers is shown in Table 1.

### 2.6. Western Blot (WB) Analysis

FaDu and FaDu/DDP cells were lysed by RIPA buffer (Beyotime Institute of Biotechnology, Shanghai, China) with protease inhibitor cocktail and phosphatase inhibitor (Thermo Fisher Scientific) and kept on ice for 15 min. After centrifugation at 15,000*g* for 25 min at 4°C, the concentration of protein was tested by BCA Protein Assay Kit (Beyotime Institute of Biotechnology). Samples with 25 *μ*g protein were separated on 8%–12% sodium dodecyl sulfate-polyacrylamide gel electrophoresis (SDS-PAGE) and transferred to polyvinylidene difluoride membrane (Merck Millipore, Darmstadt, Germany). Membranes were blocked by 5% BSA (Sigma-Aldrich, Shanghai, China) in tris-buffered saline (TBS, 10 mmol/L Tris, 10 mmol/L NaCl) for 1 h at room temperature and incubated with primary antibodies overnight at 4°C. Primary antibodies were shown as follows: GAPDH (Proteintech Group, Wuhan, China), S100A9 (Proteintech Group), CEACAM6 (Bioss, Beijing, China), IVL (Proteintech Group), IL6 (Bioss, Beijing, China), E-cadherin, and vimentin (Cell Signaling Technology, MA, USA).

### 2.7. shRNA Viral Transfection

The FaDu/DDP (4 × 10^5^) was transfected with 1 × 10^6^ transduced units of S100A9-RNAi lentivirus or negative control lentivirus (GeneChem, Shanghai, China) for 24 h. Transfection efficiency was observed by fluorescence microscope (Leica, Wetzlar, Hesse-Darmstadt, Germany). After 72 hours of transfection, cells were exposed to 5 *μ*g/ml puromycin (Thermo Fisher Scientific) for more than 2 weeks. The expression level of S100A9 was analyzed by western blot.

### 2.8. Cell Survival Assay

FaDu cells and FaDu/DDP cells were seeded at 5000/well at 96 wells plates for 24 h. The experiment group was treated with increasing concentration of cisplatin for 24 h, respectively. Thereafter, we added CCK8 reagent (Biosharp, Hefei, Anhui, China) to each well and then incubated at 37°C for 30 min. The absorbance of the culture medium was measured by Envision Microplate Reader (PerkinElmer, Waltham, Massachusetts, USA) at 450 nm.

### 2.9. Scratch Assay

Cells (50,000 cells/well) were seeded in 6-well plates and incubated overnight. Cultured with medium without FBS for 24 h and then created a straight line with a 200 *μ*l peptide tip on the cell monolayer. Washed the wells gently with PBS (HyClone, Logan, UT, USA) and incubated with FBS-free medium. The scratch region was photographed by an inverted microscope (Leica).

### 2.10. Migration Assay

Cells (75,000 cells/chamber) were cultured with 100 *μ*l FBS-free medium. The 24-well plate was added with 600 *μ*l culture medium (10% FBS) per well. After 48 h of incubation, wiped off the cells in the upper chamber. Then, the migrated cells were remained with methyl alcohol for 10 min and stained with 1% crystal violet for 30 min. The attached migrated cells were washed with PBS 3 times and then photographed and counted under an inverted microscope.

### 2.11. Proliferation Assay

Cells (5000 cells/well) were seeded in 96-well plates and cultured for 0, 24, 48, and 72 h. 10 *μ*l CCK-8 reagent was added per well and cultured in an incubator for 30 minutes. The culture medium absorbance was measured by Envision Microplate Reader at 450 nm.

### 2.12. Statistical Analysis

All experiments were performed independently in triplicate. Results are shown as means ± standard errors (SE). Datasets were analyzed by unpaired or paired *t*-tests between two groups. Statistical analysis was performed by R (version 4.1.1 for Windows), SPSS (version 22 for Windows), and GraphPad Prism (version 9.0 for Windows), and the results were considered statistically significant at *p* < 0.05.

## 3. Results

### 3.1. Different Gene Expression Analysis

**Figure 1:**
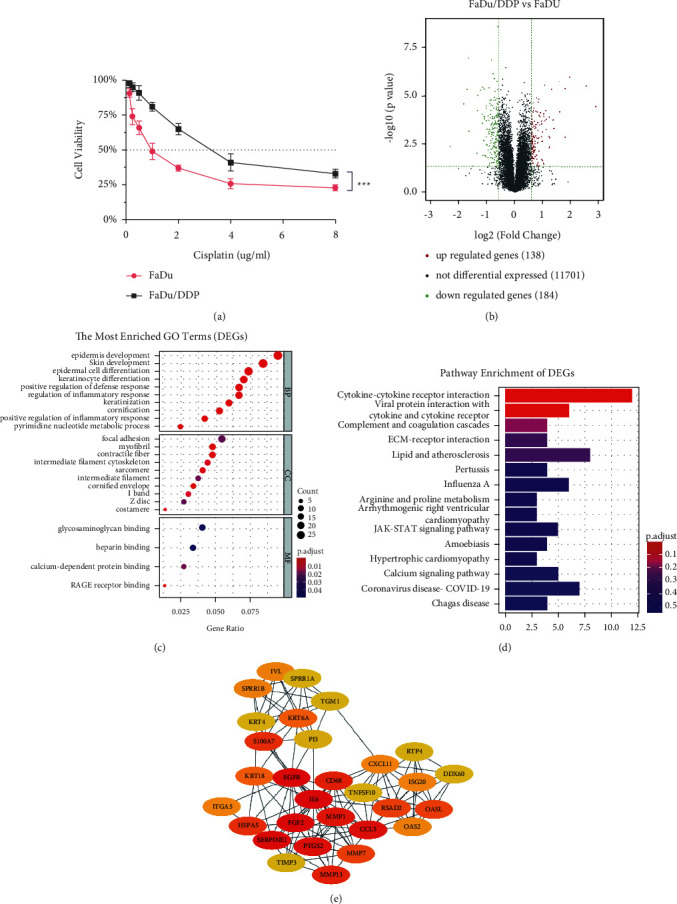
Analysis of differential expression genes in FaDu and FaDu/DDP. (a) Cisplatin-sensitivity of FaDu and FaDu/DDP cell lines. (b) DEGs volcano gram of the RNA seq. (c) GO analysis of DEGs. Top 10 enriched terms in BP, CC, and MF with a *p* value less than 0.05. (d) KEGG pathway analysis of DEGs enriched the top 15 pathways with a *p* value less than 0.05. (e) The PPI network among 30 differentially expressed cisplatin-resistance related genes.

A cisplatin-resistant FaDu cell line was induced in our research group. Before we start, we measured the cell lines' cisplatin sensibility by CCK8 assay. The IC50 of cisplatin is 1.290 ± 0.043 *μ*g/ml in FaDu and 3.708 ± 0.379 *μ*g/ml in FaDu/DDP. The FaDu/DDP cell line's cisplatin-resistance index is 2.78 (Figure 1(a)). To explore the mechanisms underlying cisplatin-resistance of HPC, we performed RNA seq analysis on both FaDu cells (*n* = 3) and FaDu/DDP cells (*n* = 3). Using bioinformatics analysis technology, 322 different expressed mRNAs (express variation >1.5, *p* value < 0.05) were screened out. Among them, 138 genes were upexpressed, and 184 genes were low expressed in FaDu/DDP compared with FaDu (Figure 1(b)). To elucidate the potential biological pathway to cisplatin resistance, we performed GO analysis on the DEGs. GO analysis divided genes into three aspects which are biological processes (BP), cellular components (CC), and molecular functions (MF). GO analysis showed that the DEGs were significantly enriched in epidermis development, skin development, epidermal cell differentiation and keratinocyte differentiation of BP, focal adhesion of CC, and calcium-dependent protein binding of MF (Figure 1(c)). Then, KEGG analysis was performed and showed that these DEGs were mainly involved in signal transmission, cell adhesion, and metabolism (Figure 1(d)). PPI network of DEGs was conducted by STRING, and the essential genes were identified by using the degree algorithm of Cytoscape's plugin cytoHubba. The nodes with darker colors in the figure are the hub genes: IL6, FGF2, SERPINE1, MMP1, and PTGS2 (Figure 1(e)). These genes could be the potential prognostic markers and therapeutic targets for overcoming cisplatin-resistance in HPC.

### 3.2. Different Protein Expression Analysis

**Figure 2:**
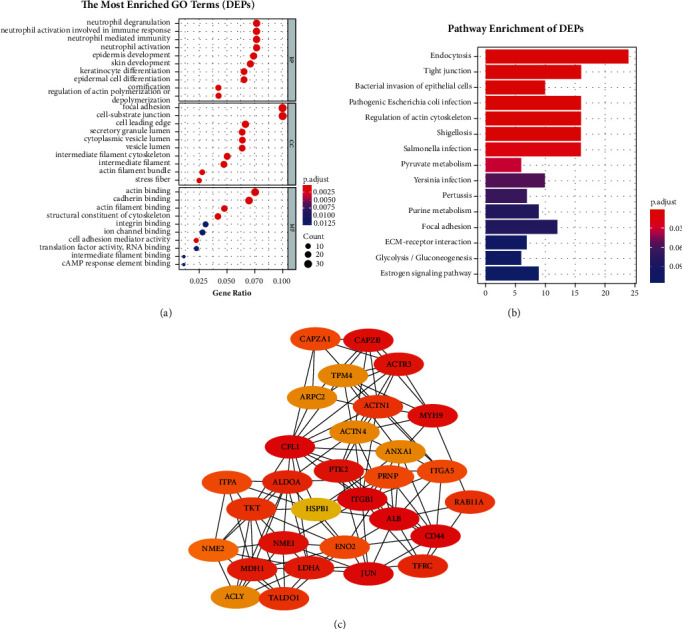
Analysis of differential expression proteins in FaDu and FaDu/DDP. (a) GO analysis of DEPs. Top 10 enriched terms in BP, CC, and MF with a *p* value less than 0.05. (b) KEGG pathway analysis of DEPs enriched the top 15 pathways with a *p* value less than 0.05. (c) The PPI network among 30 differentially expressed cisplatin-resistance-related proteins.

TMT was used to explore the DEPs that are most related to the cisplatin-resistance of HPC. 375 DEPs (express variation >1.2) were screened out between the FaDu and FaDu/DDP. The results showed that 160 proteins were low expressed, and 215 proteins were upexpressed in FaDu/DDP compared with FaDu (Supplementary Table 1). GO analysis demonstrated that the DEPs were significantly enriched in BP related to neutrophil and epidermal cells (Figure 2(a)). KEGG analysis showed that these proteins were mainly involved in endocytosis, tight junction, bacterial invasion of epithelial cells, and regulation of actin cytoskeleton (Figure 2(b)). The PPI network of DEPs identified the top 5 hub proteins, including CFL1, ACTR3, CAPZB, ARPC2, and ARPC3 (Figure 2(c)).

### 3.3. S100A9 Is a Potential Target of Cisplatin and Is Upregulated in FaDu/DDP

**Figure 3:**
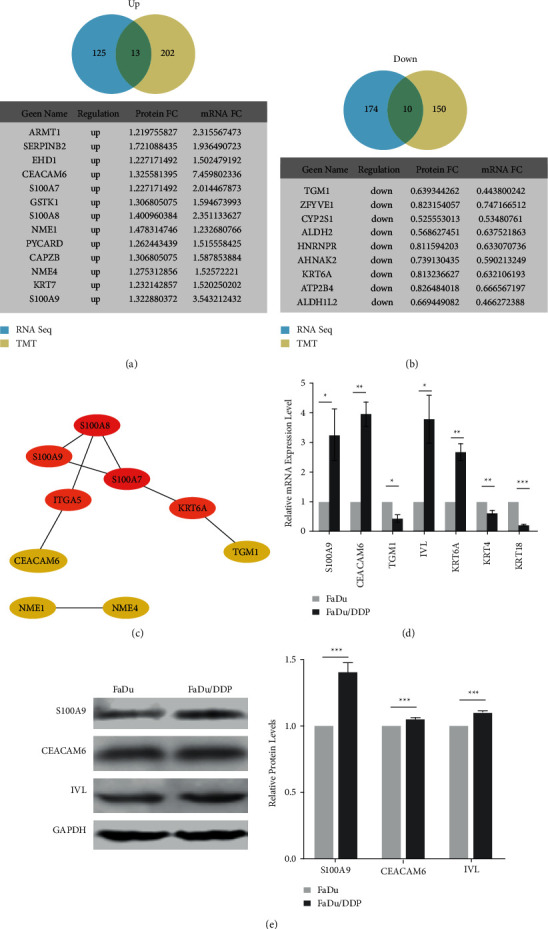
Comparison and identification the DEGs and DEPs obtained from the RNA seq and TMT data. (a) The overlapped upregulated genes between DEGs and DEPs. (b) The overlapped downregulated genes between DEGs and DEPs. (c) The PPI network of the common genes between DEGs and DEPs. (d) The mRNAs associated with cisplatin resistance were verified by qRT-PCR. (e) Western blot showed 3 differential expressed proteins levels in FaDu and FaDu/DDP. ^*∗*^*p* < 0.05, ^*∗∗*^*p* < 0.01, and ^*∗∗∗*^*p* < 0.001.

Based on the RNA sequencing and TMT results, we mapped differential expressed mRNAs and proteins and found that only 23 of them were common, 13 of them were upregulated, and 10 of them were downregulated (Figures 3(a) and 3(b)). The PPI network of overlapped DEGs and DEPs identified the top 5 hub proteins, including S100A7, S100A8, S100A9, ITGA5, and KRT6A (Figure 3(c)). We evaluated 7 genes that were involved in cell adhesion and signal transmission by RT-PCT. S100A9, IVL, and CEACAM6 were found to be significantly upregulated in RNA sequencing and TMT results and identified by RT-PCR in both cell lines. The fold change of S100A9, IVL, CEACAM6, KRT6A, TGM1, KRT4, and KRT18 in FaDu/DDP were 3.257 ± 0.504, 3.914 ± 0.363, 3.770 ± 0.464, 2.680 ± 0.159, 0.382 ± 0.057, 0.634 ± 0.037, and 0.224 ± 0.020 (Figure 3(d)). To verify the results of the analysis of transcriptome and proteome, we evaluated their expression by WB in FaDu and FaDu/DDP cell lines. The fold-change of S100A9, IVL, and CEACAM6 were 1.408 ± 0.039, 1.057 ± 0.004, and 1.110 ± 0.002. S100A9 was found to be consistently upregulated in FaDu/DDP cell line compared with the FaDu cell line (Figure 3(e)). Taking all data into consideration, we chose the S100A9 gene as a potential target of cisplatin.

### 3.4. The Migration Was Enhanced in FaDu/DDP Compared with FaDu

**Figure 4:**
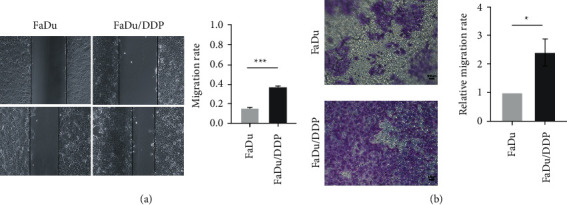
The migration phenotype in FaDu and FaDu/DDP. (a) The scratch assay showed the migration ability was promoted in FaDu/DDP compared to FaDu. (b) The migration assay showed the migration ability was promoted in FaDu/DDP compared to FaDu. ^*∗*^*p* < 0.05, ^*∗∗*^*p* < 0.01, and ^*∗∗∗*^*p* < 0.001.

GO and KEGG analysis results showed that the DEGs and DEPs were enriched in BP, such as epidermal cell differentiation and pathways, such as cell adhesion and tight junction. Our former study also mentioned that FaDu/DDP cells had residual pseudopodia [18]. We hypothesized that HPC cells with cisplatin-resistance may acquire higher migration ability. To assess the migration ability of FaDu and FaDu/DDP, we performed both scratch assay and migration assay on them. According to the scratch assay, FaDu/DDP was highly efficient in migration compared to FaDu. After 24 hours, we observed the migration rate was 15.6 ± 0.3% in FaDu and 37.3 ± 0.4% in FaDu/DDP (Figure 4(a)). Transwell assay also showed that the migration ability of FaDu/DDP was 2.400 ± 0.273 times stronger than FaDu (Figure 4(b)).

### 3.5. Low S100A9 Expression Partially Reverses Cisplatin-Resistant Phenotype and Inhibits Proliferation and Malignant Biological Properties

**Figure 5:**
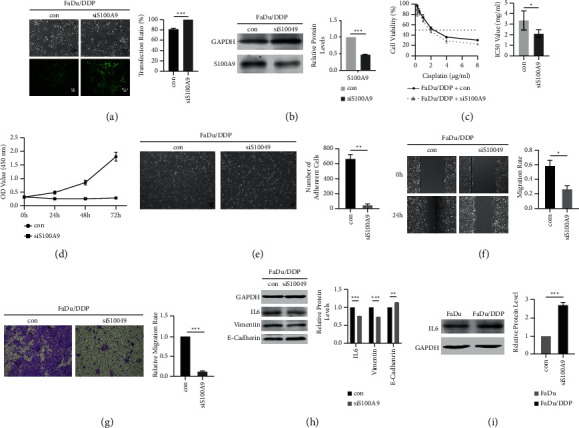
Downregulation of S100A9 reversed the cisplatin sensitivity and inhibited migration and proliferation. Con: a negative control lentivirus transfected group; siS100A9: siS100A9 lentivirus transfected group. (a) A comparison of the fluorescent and white light images after being transfected for 72 hours showed that the transfected efficiency of con and siS100A9 groups were both higher than 80%. (b) Expression of S100A9 protein was determined by western blot after lentivirus transfected and puromycin exposed for 2 weeks. (c) The downregulation of S100A9 attenuated the cisplatin sensitivity in FaDu/DDP by CCK8. (d) The proliferation assay of FaDu/DDP con and siS100A9 cell lines. (e) Number of adherent cells after lentivirus transfected for 72 hours in FaDu/DDP. (f, g) The scratch assay and migration assay showed the migration-inhibition effect of S100A9 downregulation in HPC cisplatin-resistant cells. (h) Expression of the EMT-associated proteins and IL6 protein was determined by Western blot in FaDu/DDP. (i) WB showed IL6 expression levels in FaDu and FaDu/DDP. ^*∗*^*p* < 0.05, ^*∗∗*^*p* < 0.01, and ^*∗∗∗*^*p* < 0.001.

A significantly increased expression of S100A9 in FaDu/DDP suggested that S100A9 might play an essential part in acquired cisplatin resistance. A stable S100A9 downregulation (siS100A9) construct was used to investigate the S100A9 function. We observed the fluorescence of the cells and evaluated the transfection efficiency was 81.67 ± 1.53% in FaDu/DDP con, 100% in FaDu/DDP siS100A9 (Figure 5(a)). Then, WB analysis was applied to evaluate that the protein expression patterns of S100A9. The downregulation efficiency was 51.8 ± 0.2% (Figure 5(b)). The CCK8 assay revealed that S100A9 downregulation in FaDu/DDP cell line could resensitize the FaDu/DDP cell line to cisplatin. The IC50 of cisplatin is 3.670 ± 0.095 *μ*g/ml*μ*g/ml in FaDu/DDP con and 3.708 ± 0.379 *μ*g/ml in FaDu/DDP. The FaDu/DDP siS100A9 cell line's cisplatin-resistance index is 0.624 (Figure 5(c)).

The proliferation assay showed that S100A9 downexpression inhibited cell proliferation in FaDu/DDP compared to the control group (con) (Figure 5(d)). Therefore, we were curious how downregulate S100A9 in FaDu/DDP could change its biological properties. Interestingly, we found that the adhesion ability of FaDu/DDP siS100A9 cell line was greatly reduced when we seeded and cultured the cells for 24 hours. The adherent FaDu/DDP con cells were 667 ± 52, and the adherent FaDu/DDP siS100A9 cells were 51 ± 9 (Figure 5(e)). Quantification of the wound area indicated a significantly lower scratch closure in the siS100A9 group. After 24 hours, the scratch closure was 58.9 ± 4.2% in the control group and 26.77 ± 2.61% in the siS100A9 group (Figure 5(f)). Moreover, the transwell assay also showed that low expression of S100A9 dramatically diminished the migration ability of FaDu/DDP. The relative migration rate of FaDu/DDP con and FaDu/DDP siS100A9 was 12.0 ± 1.4% (Figure 5(g)).

Furthermore, the KEGG analysis results of DEGs and DEPs both mentioned that the ECM-receptor interaction pathway might play an important role in cisplatin-resistance of HPC. Therefore, we were curious to know whether downregulate S100A9 could inhibit EMT signal pathway in FaDu/DDP. WB showed that the expression of vimentin was apparently redeemed by S100A9 downregulation, and the expression of E-cadherin was significantly increased. The fold-change of vimentin and E-cadherin were 0.734 ± 0.002 and 1.139 ± 0.008 (Figure 4(h)). EMT signal pathway was inhibited by downregulating S100A9. We also noticed that IL6 is the top 1 hub gene of DEGs' PPI network. Both IL6 and S100A9 could be boosted by inflammation. So, we speculated that IL6 could be a downstream of S100A9. We identified IL6's expression by WB, and it was found to be upregulated 2.720 ± 0.076 times in FaDu/DDP compared to FaDu (Figure 5(i)) and downregulated 0.758 ± 0.001 times in siS100A9 compared to con (Figure 5(h)). Above all, RNA sequencing and TMT results indicate that cisplatin upregulates S100A9 expression, which in turn promotes migration ability and then leads to cisplatin resistance in HPC. Downregulation of S100A9 significantly inhibits proliferation, adherence and migration abilities, redeems the cisplatin resistance of HCP, and inhibits expression of the IL6 and the EMT pathway.

## 4. Discussion

The most important goal of cancer research is identifying prognostic and therapeutic targets that are applicable to many patients. The cisplatin-based regimen is one of the first-line strategies for advanced HPC, especially for patients with recurrent or metastatic HPC. However, acquisition of chemoresistance following treatment contributes to poor survival and limits therapeutic options [4]. The problem of drug resistance in cancer is multifaceted. Previous studies showed that the addition of novel drugs with nonoverlapping mechanisms of action or more potent derivatives could result in deeper responses [27, 28]. Therefore, identifying new drug targets to overcome cisplatin-resistance of HPC is essential.

In this study, FaDu/DDP cell line was built by increasing the concentration of cisplatin exposed to FaDu. Cisplatin-resistance was detected by CCK8 assay. The IC50 of cisplatin is 1.290 ± 0.043 *μ*g/ml in FaDu and 3.708 ± 0.379 *μ*g/ml in FaDu/DDP. To identify the potential genes inducing cisplatin-resistance of HPC, RNA seq, and TMT were conducted on both cell lines. The DEGs and DEPs were screened out by R statistical programming. GO and KEGG analyses were used to enrich DEGs and DEPs. In both DEGs' and DEPs' KEGG pathway enrichment, we found “ECM-receptor interaction” was identified. We noticed that the “focal adhesion” and “tight junction” were identified in DEGs' or DEPs' KEGG pathway enrichment. In GO analysis of DEGs or DEPs, we noticed that processes of BP that related to immune regulation had emerged several times. The results revealed that extracellular matrix (ECM), cell adhesion, and immune regulation were important to the cisplatin-resistance of HPC.

We mapped DEGs and DEPs together and drew the Venn grams of up- and downregulated DEGs and DEPs. 23 genes were common, 13 were upregulated, and 10 were downregulated. Then, we selected 6 genes that were involved in cell adhesion. IVL was chosen because it was related to cell adhesion and showed upregulated in RNA seq remarkably. These 7 genes' expressions are verified by RT-PCR. According to the RT-PCR results, S100A9, CEACAM6, and IVL were remarkably upregulated. Then, WB was conducted on the 4 proteins. S100A9 was consistently upregulated in FaDu/DDP compared to FaDu. All in all, these results suggest that S100A9 is a potential target of cisplatin-resistance in HPC.

S100A9 is a member of the S100 protein family and participates in inflammatory processes and malignancies development [17]. An elevated level of S100A9 was detected in inflammation, benign tumors, and various malignancies. S100A9 has been shown to regulate proliferation, migration, and apoptosis by interacting with intracellular pathways and extracellular receptors in previous studies [29–33]. Based on previous studies and our findings, we speculated that S100A9 might be an important factor in cancer development and cisplatin-resistance of HPC. So, we conducted the CCK8 assay and demonstrated that S100A9 downregulation in FaDu/DDP reversed the cisplatin-resistance of HPC. Moreover, the cell adhesion ability was relatively lacked when we knocked down the S100A9 expression. Thus, we assumed that the function of S100A9 may relate to biological processes such as migration and proliferation. Then, we performed the proliferation, migration, and scratching assay on the siS100A9 group and con group. The results demonstrated that downregulating S100A9 inhibited the proliferation and migration ability of HPC.

To predict and identify the potential molecular and pathway vulnerabilities that could be targeted to overcome the cisplatin resistance of the HPC, we drew the PPI networks according to the DEGs and DEPs. The PPI network of DEGs found 5 hub genes: IL6, FGF2, SERPINE1, MMP1, and PTGS2, and the PPI network of DEPs found 5 hub proteins: CFL1, ACTR3, CAPZB, ARPC2, and ARPC3. We noticed the IL6 gene in the core of the PPI network of DEGs. IL6 is a pluripotent molecule involved in immune regulation and inflammatory response [34]. Previous studies have demonstrated that S100A9 has a positive regulatory effect on IL6 in inflammatory [31, 35]. Furthermore, a series of studies in recent years also pointed out that IL6 might be a critical factor in drug resistance of various tumors [36–38]. We were curious whether the cisplatin and downregulation of S100A9 could affect the IL6 expression. Then, we identified that both cisplatin and the downregulation of S100A9 could inhibit the expression of IL6. It reveals that IL6 may play as the downstream target of S100A9.

Zha et al. found that S100A9 promoted the proliferation and migration of cervical cancer. Besides, epithelial marker E-cadherin was suppressed, and mesenchymal marker vimentin was increased [32]. EMT is a process in which epithelial cells lose their ability of polarization and cell adhesion and gain the mesenchymal stem cell phenotype, such as proliferation, migration, and drug resistance [39]. As GO and KEGG analyses have mentioned, ECM and cell adhesion were important in the cisplatin-resistance of HPC. Proliferation, migration, and CCK8 assay demonstrated that downregulation of S100A9 in FaDu/DDP cells could inhibit the ability of proliferation, migration, and cisplatin-resistance. WB identified E-cadherin was upregulated, and vimentin was downregulated when we knocked down S100A9 in FaDu/DDP. The above results suggested that the downregulation of S100A9 could suppress the mesenchymal properties of HPC, which might result in the suppression of the EMT pathway.

In recent years, molecular target therapy and immunotherapy have been developed to overcome head and neck cancers. However, whether the addition of these drugs to chemoradiotherapy has a beneficial effect on HNSCC patients is still controversial [40, 41]. A single-center clinical trial demonstrated that additional use of nimotuzumab (anti-EGFR) could benefit advanced HPSCCa patient population with tolerable toxicity, but two multicenter clinical trials of avelumab (anti-PD-L1) and lapatinib (EGFR/ErbB2 inhibitor) in the HNSCC patient population, respectively, showed opposite results [42–44]. Therefore, identifying more drug targets to improve the treatment's effect and safety for cisplatin-resistant HPC patients is still particularly in need. Goh et al. found calcium-binding proteins S100A7, S100A8, and S100A9 built a reciprocal feedback loop with IL-1receptor-associated kinase 1 (IRAK1). Besides, pacritinib, a kinase inhibitor with specificity for IRAK1, can disrupt the loop [45]. Moreover, Liu et al. demonstrated that downregulation of IRAK1 reversed the paclitaxel-resistance in nasopharyngeal carcinoma, and combined treatment of pacritinib with paclitaxel improved antitumor effect [46]. Pacritinib is a potential molecular target therapy that can be used in HPC patients [47]. However, whether the blockade of IRAK1 and pacritinib could overcome cisplatin resistance in HPC still need our following research.

In conclusion, our study demonstrated that downregulation of S100A9 remarkably inhibited the migration and proliferation and reversed resistance of HPC to cisplatin. We noticed that IL6 and EMT pathway might be downstream of S100A9. However, these results were based on bioinformatic analysis and in vitro studies. More particular and in vivo studies will complement in the future.

## Figures and Tables

**Table 1:** Sequence of primers for RT-PCR.

Gene	Primer	Sequence
S100A9	Forward	5′-TCCTCGGCTTTGACAGAGTG-3′
	Reverse	5′-GTCACCCTCGTGCATCTTCT-3′

CEACAM6	Forward	5′-ACCCTGAATGTCCTCTATGGC-3′
	Reverse	5′-GAGAGGACAGGAGCACTTCC-3′

TGM1	Forward	5′-CTCTGGCACTCGAAGACCTG-3′
	Reverse	5′-TACTAGCATGCCCTCTCGGA-3′

IVL	Forward	5′-AGGCCCTCAGATCGTCTCAT-3′
	Reverse	5′-CTGAGGTTGGGATTGGGGTC-3′

KRT6A	Forward	5′-TGGACAAGTCAACATCTCTGTGG-3′
	Reverse	5′-ACCGAGAGCTAGCAGACGC-3′

KRT4	Forward	5′-GAGGGCGAGGAGTACAGAATG-3′
	Reverse	5′-CCCGGAGCCACTTCCTAATC-3′

KRT18	Forward	5′-CCTACAAGCCCAGATTGCCA-3′
	Reverse	5′-TGGTGCTCTCCTCAATCTGC-3′

GAPDH	Forward	5′-GTGTTCCTACCCCCAATGTG-3′
	Reverse	5′-AGGAGACAACCTGGTCCTCA-3′

## Data Availability

The datasets generated during and/or analyzed during the current study are available from the corresponding author on reasonable request.
